# Gestational Age, Infant Birth Weight, and Subsequent Risk of Type 2 Diabetes in Mothers: Nurses’ Health Study II

**DOI:** 10.5888/pcd10.120336

**Published:** 2013-09-19

**Authors:** Tamarra M. James-Todd, S. Ananth Karumanchi, Eileen L. Hibert, Susan M. Mason, Mary A. Vadnais, Frank B. Hu, Janet W. Rich-Edwards

**Affiliations:** Author Affiliations: S. Ananth Karumanchi, Mary A. Vadnais, Howard Hughes Medical Institute, Beth Israel Deaconess Medical Center, and Harvard Medical School, Boston, Massachusetts; Eileen L. Hibert, Susan M. Mason, Brigham and Women’s Hospital, and Harvard Medical School, Boston, Massachusetts; Frank B. Hu, Harvard School of Public Health, Boston, Massachusetts; Janet W. Rich-Edwards, Brigham and Women’s Hospital, Harvard Medical School, and Harvard School of Public Health, Boston, Massachusetts.

## Abstract

**Introduction:**

Women with a history of gestational diabetes mellitus (GDM) are at higher risk of developing type 2 diabetes (T2DM); however, little is known about the association between other common pregnancy complications (eg, preterm birth, macrosomia) and T2DM risk. We examined the associations between first-pregnancy preterm, postterm birth, low birth weight, and macrosomia with subsequent risk of T2DM.

**Methods:**

We conducted a prospective cohort study of Nurses’ Health Study II (NHSII) participants; 51,728 women in the study had a single live birth and complete pregnancy history. NHSII confirmed incident diabetes mellitus through supplemental questionnaires. Participants were followed from year of first birth until 2005. We defined gestational age as very preterm (20 to ≤32 weeks), moderate preterm (33 to ≤37 weeks), term (38 to ≤42 weeks), and postterm (≥43 weeks). We defined low birth weight as an infant born at term weighing less than 5.5 pounds, and we defined macrosomia as an infant born at term weighing 10 pounds or more. We used Cox proportional hazards models, adjusting for potential confounders.

**Results:**

Women with a very preterm birth (2%) had an increased T2DM risk (adjusted hazard ratio, 1.34; 95% confidence interval [CI], 1.05–1.71). This increased risk emerged in the decade following pregnancy. Macrosomia (1.5%) was associated with a 1.61 increased T2DM risk, after adjusting for risk factors, including GDM (95% CI, 1.24–2.08). This association was apparent within the first 5 years after pregnancy. Moderate preterm and term low birth weight did not significantly increase the risk of T2DM over the 35-year follow-up time.

**Conclusion:**

Women who experienced a very preterm birth or had an infant that weighed 10 pounds or more may benefit from lifestyle intervention to reduce T2DM risk. If replicated, these findings could lead to a reduced risk of T2DM through improved primary care for women experiencing a preterm birth or an infant of nonnormal birth weight.

## Introduction

A growing body of research suggests that pregnancy and the period surrounding it may provide unique information about a woman’s future risk of chronic disease (1,2). For example, gestational diabetes (GDM) is a well-established risk factor for type 2 diabetes (T2DM) in women (3,4). Although up to 70% of women who develop GDM will eventually develop T2DM within the first 5 to 20 years following pregnancy (5), several studies have shown that lifestyle interventions immediately following pregnancy lead to a significant reduction in T2DM risk (6,7).

Despite the well-established association between GDM and future risk of T2DM (5,8), less is known about even more common pregnancy complications and future risk of T2DM. For example, preterm birth and low birth weight complicate more than 10% of US pregnancies (9,10). Macrosomia occurs in 1% to 10% of all pregnancies. Preterm birth and low birth weight share common underlying risk factors with T2DM, including elevated pre-pregnancy and pregnancy lipid concentrations (11,12) and inflammatory markers (13,14). These shared biological factors suggest that preterm birth and low term birth weight may be early markers of subclinical risk of future development of T2DM. In addition, giving birth to a macrosomic infant (10 pounds or more) could suggest an increased risk of future maternal T2DM in the absence of GDM or could result from undiagnosed GDM. For example, T2DM could result from excess glucose exposure and consequent high fetal growth from impaired glucose tolerance that falls short of GDM diagnostic criteria (15). If such associations are found, women experiencing these complications could potentially benefit from early intervention to reduce future T2DM risk.

The objective of our study was to examine the association between gestational age, birth weight, and T2DM in mothers. We evaluated these associations in a large cohort study, adjusting for potential confounders, including maternal and paternal history of diabetes, pre-pregnancy body mass index (BMI), and smoking during pregnancy. We also adjusted for lifestyle and reproductive factors as well as pregnancy complications known to be predictors of T2DM. Finally, we explored the time trends in risk over the decades following birth of a preterm infant or an infant of nonnormal birth weight to suggest potential windows for prevention and glucose tolerance screening after complicated pregnancies.

## Methods

### Study population

Study participants were from the Nurses’ Health Study II (NHSII) population, a cohort study of 116,678 female nurses who were aged 25 to 42 years at the start of the study in 1989. NHSII follows participants biennially by questionnaire to obtain both health-related behavior information and data on the occurrence of diseases, including diabetes. The follow-up rate has been approximately 90% for each biennial questionnaire. For purposes of this study, we followed women from their first pregnancy (the earliest pregnancy in our study population occurred in 1964) through 2005, the last reported follow-up for incident diabetes.

**Figure 1:**
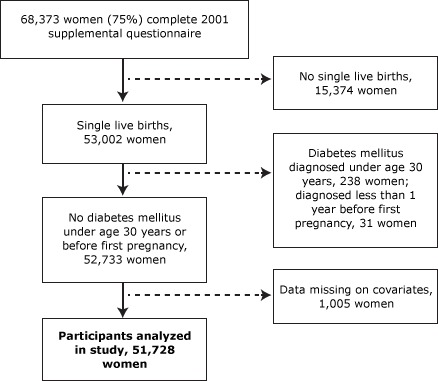
Characteristics of the study population, 2001 Nurses’ Health Study II supplemental questionnaire.

Our study population is based on a subset of the NHSII population who had detailed data on pregnancy history and T2DM. More specifically, in 2001, NHSII sent a supplemental questionnaire to study participants who were deemed good responders — those women who typically responded to the first or second mailing of the biennial questionnaires. This supplemental questionnaire included detailed data on history of pregnancies that lasted at least 12 weeks; 68,376 women (75% of those mailed the supplemental questionnaire and 58% of the original study population) returned the questionnaire (Figure 1). We excluded women with twins or triplets, women who had had more than 5 pregnancies (the questionnaire length precluded collection of detailed pregnancy data for more than 5 pregnancies), and women whose questionnaires were missing data on gestational age or birth weight or were missing covariate data (Figure 1). We also excluded women who were diagnosed with diabetes at or before age 30 to avoid including those with type 1 diabetes mellitus and women who were diagnosed with diabetes mellitus before their first pregnancy. This study was approved by The Partners Human Research Committee (institutional review board) of Brigham and Women’s Hospital.

### Pregnancy complications

Study participants indicated the length of each of their 5 most recent pregnancies lasting at least 12 weeks in the following categories: 12 to less than 20 weeks, 20 to less than 24 weeks, 24 to less than 28 weeks, 28 to less than 32 weeks, 32 to less than 37 weeks, 37 to 42 weeks, and 43 or more weeks. Pregnancies were categorized as very preterm (20 to ≤32 weeks), moderate preterm (33to ≤37 weeks), term (37 to ≤42 weeks, referent) and postterm (≥43 weeks).

Participants were asked the birth weight for each of their 5 most recent pregnancies (<5 lbs, 5–5.4 lbs, 5.5–6.9 lbs, 7.0–8.4 lbs, 8.5–9.9 lbs, ≥10 lbs). Low birth weight was defined as less than 5.5 pounds at term and macrosomia as 10 pounds or more at term.

### Gestational diabetes

In the NHSII 1989 baseline questionnaire and subsequent biennial questionnaires study participants indicated whether they had ever been diagnosed with GDM by a physician. A validation of GDM showed high validity of self-report of this condition compared with medical records (16).

### Hypertensive disorders of pregnancy

Study participants responded to “Have you ever had toxemia/pre-eclampsia (raised blood pressure and proteinuria) with any pregnancy?” on the NHSII baseline questionnaire and biennially. A recent validation of pre-eclampsia compared with medical record review showed self-reported preeclampsia is a moderately good indicator of hypertensive disorders of pregnancy (HDOP) (positive predictive value, 73%) (17).

### Type 2 diabetes

NHSII study participants received a supplemental questionnaire if they reported having been diagnosed with diabetes mellitus on the biennial questionnaires between 1991 and 2005. These supplemental questionnaires collected information to distinguish between type 1 and type 2 diabetes mellitus on the basis of diabetes diagnosis and treatment. The validation of this method of case confirmation has been reported previously (18). The 6% of respondents who reported having diabetes on the baseline 1989 questionnaire were not sent the supplemental questionnaire and, consequently, had details only on date of diagnosis, without information on whether the diabetes was type 1 or type 2. To restrict the analysis to cases likely to be T2DM, we included women who reported having diabetes mellitus on the 1989 questionnaire only if they were age 30 years or older at the time of diagnosis.

### Lifestyle factors and medical history

On the 1989 baseline questionnaire, study participants reported their current weight and height and their weight at age 18 (19). Participants were asked to update their weight on all biennial questionnaires. Body mass index (BMI, kg/m^2^) was calculated from reported height and weight data. BMI was derived for ages at which weight was not reported (ie, between age 18 and age at the baseline questionnaire) from a formula using weight at age 18 and weights reported on biennial questionnaires, as well as somatograms at ages 20, 30, and 40 to assign BMI at each age starting at 18 years and through the end of the study period.

Participants reported their race/ethnicity at baseline. Subsequent biennial questionnaires queried family history of diabetes mellitus, as well as a personal medical history (eg, hypertension, cancer, gestational diabetes, pre-eclampsia, toxemia). In addition, the 2001 supplemental questionnaire asked about smoking status during pregnancy (eg, ever, never). Participants were also asked at baseline to report their menstrual regularity at age 18 to 22 years, which was categorized as regular, irregular, or no menstrual periods.

### Statistical analysis

We used Cox proportional hazards models to examine the association between preterm and postterm birth, as well as low birth weight and macrosomia with T2DM risk, modeling gestational age and birth weight separately. Very preterm birth, moderate preterm birth, and postterm birth were evaluated as indicator variables, with the reference group of term. For the association between birth weight and T2DM, we did not have enough information on birth weights under 5.5 pounds to create small-for-gestational-age categories among preterm births. Therefore, we restricted our analysis to women who had a term birth (n = 42,502). Models were constructed among all term births by using indicator variables for low birth weight, macrosomia, and normal birth weight (reference group).

Women were followed from their first birth through 2005 or up to 35 years after their first pregnancy; we were able to follow more than 95% of the cohort for up to 34 years after their first birth. First, we evaluated the association between gestational age, infant birth weight, and T2DM risk in first pregnancies for the entire 35-year follow-up period. Second, we explored time since first birth in 5-year intervals up to 35 years after the first pregnancy to determine at what time points the differences between gestational age or birth-weight groups were significant. Hazard ratios (HRs) and 95% confidence intervals (CIs) were calculated. We adjusted for potential confounders, those factors that preceded pregnancy complications of interest and were associated with diabetes mellitus. Finally, we used the Breslow estimator to calculate the age-adjusted risk differences from the Cox proportional hazards models. All analyses were conducted using SAS statistical software (version 9.3; SAS Institute, Inc, Cary, North Carolina).

## Results

In our study population of NHSII participants, births occurred between 1964 and 2001, with 95% occurring before 1993. We examined pregnancy complications and risk factors for all births and for term births in the study population (Table 1).

**Table 1:** Pregnancy Complications and Diabetes Risk Factors, Nurses’ Health Study II, 1989–2005

Characteristic	All Births					Term Births		
	Total Births	Very Preterm	Moderate Preterm	Term	Postterm	Low Birth Weight	Normal Birth Weight	Macro- somia
Total no. (%)	51,728 (100)	1,088 (2.1)	3,460 (6.7)	42,502 (82.2)	4,678 (9.0)	554 (1.3)	41,294 (97.2)	654 (1.5)
Age at first birth, mean (SD)	27.1 (4.7)	27.3 (5.4)	27.7 (4.8)	27.1 (4.7)	26.6 (4.5)	26.9 (5.0)	27.1 (4.6)	27.6 (4.5)
Age at 1989 baseline, mean (SD)	34.6 (4.7)	35.4 (4.5)	34.1 (4.7)	34.5 (4.7)	35.4 (4.5)	35.8 (4.6)	34.5 (4.7)	34.0 (4.7)
BMI at 1st pregnancy, mean (SD)	22.1 (3.1)	22.3 (3.6)	22.2 (3.2)	22.1 (3.1)	22.3 (3.3)	21.9 (2.9)	22.0 (3.0)	23.8 (4.4)
BMI in 1989, mean (SD)	23.8 (4.6)	24.2 (4.9)	23.5 (4.5)	23.7 (4.5)	24.7 (5.3)	23.7 (4.6)	23.7 (4.5)	25.7 (5.6)
White, n (%)	48,292 (93.4)	999 (91.8)	3,171 (91.7)	39,705 (93.4)	4,417 (94.4)	505 (91.2)	38,577 (93.4)	623 (95.3)
Family history of diabetes^a^, n (%)	15,457 (29.9)	367 (33.7)	1,127 (32.6)	12,512 (29.4)	1,451 (31.0)	175 (31.6)	12,125 (29.4)	212 (32.4)
GDM status, n (%)	2,882 (5.6)	86 (7.9)	245 (7.1)	2,280 (5.4)	271 (5.8)	27 (4.9)	2,164 (5.2)	89 (13.6)
HDOP status, n (%)	7,653 (14.8)	219 (20.1)	841 (24.3)	5,824 (13.7)	769 (16.4)	135 (24.4)	5,560 (13.5)	129 (19.7)
Smoking during pregnancy, n (%)	7,104 (13.7)	178 (16.4)	452 (13.1)	5,727 (13.5)	747 (16.0)	132 (23.8)	5,534 (13.4)	61 (9.3)
Irregular or no menstrual periods at age 18–22, n (%)	11,941 (23.1)	305 (28.0)	790 (22.8)	9,550 (22.5)	1,296 (27.7)	120 (21.7)	9,297 (22.5)	133 (20.3)

Abbreviations: SD, standard deviation; BMI, body mass index; GDM, gestational diabetes mellitus; HDOP, history of hypertensive disorders of pregnancy.

a Participant’s mother or father diagnosed with diabetes.

### Gestational age and type 2 diabetes

In age-adjusted analysis, we observed a graded association of very and moderate preterm birth with risk of T2DM, as well as elevated risks among women delivering their first child postterm (Table 2). However, adjustment for age at first birth, age in 1989, race/ethnicity, pre-pregnancy BMI, family history of diabetes mellitus, menstrual irregularity, smoking status, history of GDM, and history of HDOP dampened several of these associations; very preterm birth and postterm birth conferred an increased risk of T2DM, and moderate preterm birth no longer showed a significant increased risk of T2DM.

**Table 2:** Association Between First Birth Gestational Age and Type 2 Diabetes, Nurses’ Health Study II, 1989–2005

Birth	N (%)	Age-Adjusted Hazard Ratio (95% Confidence Interval)^a^	Adjusted Hazard Ratio (95% Confidence Interval)^b^
Very preterm	1,088 (2.1)	1.65 (1.30–2.11)	1.34 (1.05–1.71)
Moderate preterm	3,460 (6.7)	1.34 (1.13–1.58)	1.12 (0.95–1.32)
Term	42,502 (82.2)	Reference	Reference
Postterm	4,678 (9.0)	1.29 (1.13–1.48)	1.15 (1.00–1.32)

a Adjusted for age in 1989.

b Adjusted for maternal age at first birth, age in 1989, race/ethnicity, pre-pregnancy body mass index, family history of diabetes, menstrual irregularity, smoking during pregnancy, history of gestational diabetes mellitus, history of hypertensive disorders of pregnancy.

**Figure 2:**
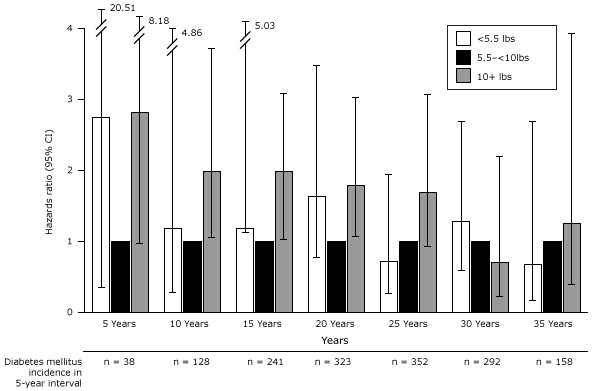
Association between gestational age and incidence of type 2 diabetes by 5-year intervals following first pregnancy adjusted for age at first birth, age at baseline Nurses’ Health Study II, race/ethnicity, pre-pregnancy body mass index, family history of diabetes, menstrual irregularity, smoking during pregnancy, gestational diabetes, and hypertensive disorders of pregnancy. Abbreviations: CI, confidence interval; NA, not applicable. **Table:** 

We explored the association between gestational age and T2DM in 5-year intervals following first pregnancy (Figure 2). In the first decade after very preterm birth, there was no increased risk of T2DM compared with term birth. However, there was an inconsistent change in risk in the second decade, which reached statistical significance. No significant associations were found after 21 years following first pregnancy among women with a very preterm birth. In contrast, women who had a moderate preterm birth had significant, roughly two-fold, increased risk of T2DM for the first 10 years after their first pregnancy, which thereafter returned to the baseline risk of women who had delivered at term. Postterm birth did not appear to be associated with T2DM at any time period.

Total Number of Diabetes Cases in 5-Year Intervals	Adjusted Hazard Ratio (95% Confidence Interval)
5 years, n = 52	
Very preterm, n = 2	0.93 (0.22–3.93)
Moderate preterm, n = 9	1.96 (0.93–4.13)
Term, n = 38	1 [Reference]
Postterm, n = 3	1.00 (0.30–3.29)
10 years, n = 175	
Very preterm, n = 5	0.89 (0.36–2.18)
Moderate preterm, n = 26	1.82 (1.19–2.80)
Term, n = 128	1 [Reference]
Postterm, n = 16	1.19 (0.71–2.01)
15 years, n = 314	
Very preterm, n = 14	1.70 (0.99–2.92)
Moderate preterm, n = 27	1.13 (0.76–1.69)
Term, n = 241	1 [Reference]
Post term, n = 32	1.07 (0.74–1.55)
20 years, n = 416	
Very preterm, n = 8	0.70 (0.34–1.41)
Moderate preterm, n = 31	0.98 90.68–1.42)
Term, n = 323	1 [Reference]
Postterm, n = 54	1.23 (0.92–1.64)
25 years, n = 456	
Very preterm, n = 17	1.57 (0.96–2.56)
Moderate preterm, n = 29	1.00 (0.69–1.47)
Term, n = 352	1 [Reference]
Postterm, n = 58	1.14 (0.86–1.51)
30 years, n = 375	
Very preterm, n = 14	1.67 (0.97–2.84)
Moderate preterm, n = 20	0.90 (0.57–1.41)
Term, n = 292	1 [Reference]
Postterm, n = 49	1.19 (0.88-1.61)
35 years, n = 204	
Very preterm, n = 8	1.79 (0.88–3.65)
Moderate preterm, n = 14	1.32 (0.76–2.29)
Term, n = 158	1 [Reference]
Postterm, n = 24	1.16 (0.75-–.79)

We also explored the findings using risk differences. We found that during the 6 to 10 years following the first pregnancy, only 12 excess T2DM cases per 10,000 women who experienced a moderate preterm birth occurred compared with women who delivered at term. By 26 to 30 years after the first pregnancy, 298 excess T2DM cases per 10,000 women who delivered a very preterm birth occurred compared with those who delivered at term.

### Birth weight and type 2 diabetes

Although term low birth weight was not associated with T2DM in the overall population (fully adjusted HR, 1.26; 95% CI, 0.88–1.81), an increased risk for women who delivered a term low birth weight infant was seen among those without a history of GDM or HDOP (fully adjusted HR, 1.62; 95% CI, 1.01–2.59). Women who gave birth to a first infant that weighed 10 pounds or more had a 1.61 increased risk of T2DM (95% CI, 1.24–2.08) (Table 3). Restricting the results to women without a history of GDM or HDOP somewhat strengthened the association (fully adjusted HR, 2.22; 95% CI, 1.50–3.28).

**Table 3:** Association Between First Pregnancy Birth Weight and Type 2 Diabetes Among Women with Term Births, Nurses’ Health Study II, 1989–2005

Term Birth Weight	N (%)	Age-adjusted Hazard Ratio (95% CI)^a^	Adjusted Hazard Ratio (95% CI)^b^
Term low birth weight	554 (1.3)	1.40 (0.98–2.01)	1.26 (0.88–1.81)
Term normal birth weight	41,294 (97.2)	1 [Reference]	1 [Reference]
Term macrosomia	654 (1.5)	2.96 (2.29–3.82)	1.61 (1.24–2.08)

Abbreviation: CI, confidence interval.

a Adjusted for age in 1989.

b Adjusted for maternal age at first birth, age in 1989, race/ethnicity, pre-pregnancy body mass index, family history of diabetes, menstrual irregularity, smoking during pregnancy, history of gestational diabetes mellitus, history of hypertensive disorders of pregnancy.

**Figure 3:**
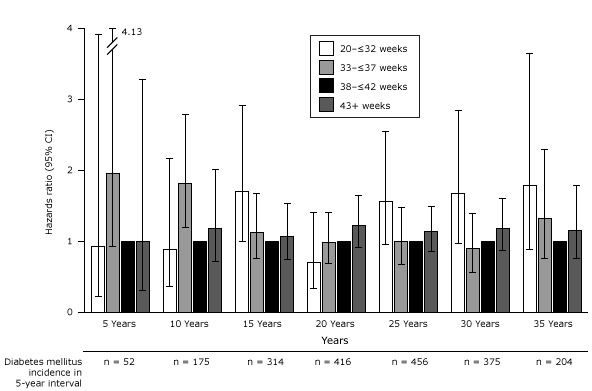
Association between birth weight and type 2 diabetes among term births by 5-year intervals following first pregnancy adjusted for age at first birth, age at baseline Nurses’ Health Study II, race/ethnicity, pre-pregnancy body mass index, family history of diabetes, menstrual irregularity, smoking during pregnancy, gestational diabetes, and hypertensive disorders of pregnancy.. Abbreviations: CI, confidence interval; NA, not applicable. **Table:** 

In our exploratory analysis evaluating time trends in risk of T2DM by 5-year intervals since first birth, we found women who had a term, low birth weight infant had an inconsistently elevated risk of developing T2DM within the first 2 decades after the first pregnancy (Figure 3); this association reached significance only at 11 to 15 years after delivery (adjusted HR, 2.36; 95% CI, 1.11–5.03). Macrosomia also conferred a large but decreasing risk of T2DM. Associations between macrosomia and subsequent risk of T2DM were only significant between 6 and 20 years after the first pregnancy.

Total Number of Diabetes Cases in 5-Year Intervals	Adjusted Hazard Ratio (95% Confidence Interval)
5 years, n = 38	NA
Term low birth weight, n = 1	2.75 (0.37–20.51)
Term normal birth weight, n = 33	1 [Reference]
Macrosomia, n = 4	2.82 (0.97–8.18)
10 years, n = 128	NA
Term low birth weight, n = 2	1.19 (0.29–4.86)
Term normal birth weight, n = 115	1 [Reference]
Macrosomia, n = 11	1.99 (1.06–3.75)
15 years, n = 241	NA
Term low birth weight, n = 7	2.36 (1.11–5.03)
Term normal birth weight, n = 220	1 [Reference]
Macrosomia, n = 14	1.79 (1.03–3.10)
20 years, n = 323	NA
Term low birth weight, n = 7	1.64 (0.78–3.49)
Term normal birth weight, n = 301	1 [Reference]
Macrosomia, n = 15	1.79 (1.06–3.03)
25 years, n = 352	NA
Term low birth weight, n = 4	0.72 (0.27–1.94)
Term normal birth weight, n = 337	1 [Reference]
Macrosomia, n = 11	1.69 (0.93–3.09)
30 years, n = 292	NA
Term low birth weight, n = 7	1.28 (0.60–2.71)
Term normal birth weight, n = 282	1 [Reference]
Macrosomia, n = 3	0.71 (0.23–2.22)
35 years, n = 158	NA
Term low birth weight, n = 2	0.67 (0.17–2.71)
Term normal birth weight, n = 153	1 [Reference]
Macrosomia, n = 3	1.25 (0.40–3.95)

When evaluating absolute risk of T2DM for women who delivered an infant that weighed 10 pounds or more, we found an excess of 115 T2DM cases per 10,000 women in this group compared with women who delivered a normal weight infant in the 21 to 25 years following the first pregnancy. After 30 years, the elevated risk associated with having delivered a large infant had disappeared.

Because the prevalence of screening for GDM and HDOP during pregnancy has changed over the period of the births in this cohort (1964–2001), we repeated the analysis among first births occurring after the baseline 1989 questionnaire, because after that time period, screening was more widely practiced as standard care and gestational age-dating of pregnancies had probably improved with the advent of ultrasound. The hazard ratios estimated by the fully adjusted models (including GDM and HDOP diagnoses) were stronger for birth weight and T2DM, which mitigated the concern that macrosomia in the earlier pregnancies might merely be a marker for undiagnosed GDM. However, the association of very preterm delivery with T2DM was no longer detectable when limiting the analysis to pregnancies occurring after 1989.

## Discussion

In this study, the approximately 9% of women whose first infant was delivered preterm had excess risk of developing T2DM, even after accounting for potential medical and lifestyle confounders. The 2% of women who experienced a very preterm birth had a 34% increased risk of developing T2DM over the 35-year follow-up period. In an exploratory analysis, we found that the elevation in risk first became evident at 11 to 15 years after the first pregnancy. Postterm birth was associated with a slight, significant increase in risk of T2DM over the entire 35-year period. A history of having borne a first infant who was term low birth weight or macrosomic conferred an almost 2 to 3-fold increased risk of T2DM, which gradually waned over time. These findings held even after adjusting for GDM and HDOP, known predictors of T2DM (8,20–22). If our findings are replicated in future studies, gestational age and offspring birth weight may be useful to identify high-risk women. Our exploratory analysis , if replicated, may also provide information on time windows in which glucose tolerance screening might be an effective addition to the primary care of high-risk women.

In a previous study by Lykke et al based on vital statistics registry data from Denmark (23), preterm birth (<37 weeks) was associated with a 2-fold increased risk of T2DM in mothers after adjusting for maternal age, year of delivery, and pregnancy complications. The median study follow-up time was approximately 15 years. Unlike our study, no adjustment was made for pre-pregnancy risk factors, such as overweight/obesity and family history, strong predictors of T2DM. Another study by Catov et al found 76% increased odds of metabolic syndrome among women with a previous preterm birth 8 years following pregnancy (24). We observed a very similar 2-fold increased risk of T2DM after a moderate preterm delivery (<37 weeks) in the first 10 years after pregnancy. However, we were able to follow our cohort for an additional 20 years, which indicated that the excess risk associated with a history of moderate preterm birth was limited to the first 10 years after pregnancy. The risk associated with very preterm birth, however, arose after the first decade following the first pregnancy and remained modestly elevated throughout the 35 years of follow-up. However, the weakening over time of relative risk associated with preterm delivery and infant birth weight may reflect the increasing prevalence of other T2DM risk factors with increasing age (study time), including high BMI and increased sedentary behaviors. Therefore, our findings may suggest that these pregnancy complications may be especially useful predictors of early-onset T2DM.

The increased risk of T2DM among women who experience a preterm birth may be due to chronic low-level inflammation (25,26). Several studies suggest that chronic low-level inflammation precedes the onset of T2DM (26–28). As such, preterm birth could signal a chronic state of inflammation and an increased risk of future development of T2DM. In addition, the association between macrosomia and T2DM, independent of GDM status, could be attributed to maternal hyperglycemia, which is less overt than GDM and can lead to fetal hyperglycemia, exaggerated fetal insulin response, and macrosomia. Therefore, macrosomia could simply indicate hyperglycemia in mothers, despite not meeting clinical definitions for a GDM diagnosis. In fact, the Hyperglycemia and Adverse Pregnancy Outcomes (HAPO) study found a continuous association between maternal hyperglycemia and increasing birth weight (15).

Strengths of this study include use of a large cohort of nurses with detailed information on both pregnancy history and diabetes and information on pre-pregnancy and reproductive risk factors for diabetes. We were also able to control for GDM and HDOP as strong predictors of T2DM. In addition, this study had an average follow-up time after first birth of 22 years, which allowed for sufficient time for a substantial proportion of participants to develop the disease (approximately 4% of the population). Furthermore, we were able to explore these research questions by using different cut points in total study time. This technique allowed us to explore periods in which certain pregnancy complications may have the most predictive value for future development of T2DM. Future studies are needed to further explore and confirm these associations based on time since pregnancy.

Our study has several limitations. First, data on gestational age and birth weight were unvalidated self-reports. However, validation studies demonstrated good self-report of related pregnancy factors (16,19). Also, several validation studies show moderate to high reliability of self-report of preterm birth and infant birth weight when compared with medical records (29–31). Second, we used categories of gestational age and birth weight instead of continuous measures, which may make it difficult to see subtle changes in disease risk. Furthermore, these categorizations prevented us from examining small-for-gestational age or large-for-gestational-age infants; as such we had to restrict our analysis of birth weight to term births. Thus, our finding of an early elevation in T2DM risk for women who delivered infants that were term low birth weight may not be generalizable to women who delivered preterm infants, who were small for gestational age. We also had limited power to examine recurrent complications in later pregnancies. Finally, we had limited ability to evaluate this association among minorities, who have both a higher prevalence of these pregnancy complications and T2DM.

Women who experience a preterm birth or have an infant with nonnormal birth weight are not followed up for lifestyle intervention or disease prevention after re-entry into the standard health care system for nonpregnant women. Both the American Diabetes Association and American College of Obstetrics and Gynecology recommend screening for T2DM for women with a history of GDM (32,33). If our findings are replicated, women who experience a preterm birth or have a nonnormal birth-weight infant may benefit from additional follow-up and lifestyle intervention to reduce their subsequent risk of T2DM.
